# Prevalence and risk factors of diabetic retinopathy in Basrah, Iraq

**DOI:** 10.25122/jml-2022-0170

**Published:** 2023-02

**Authors:** Mohammed Al Ashoor, Ali Al Hamza, Ibrahim Zaboon, Ammar Almomin, Abbas Mansour

**Affiliations:** 1 Department of Ophthalmology, Al Zahraa Medical College, University of Basrah, Basrah, Iraq; 2 Department of Ophthalmology, Basrah Teaching Hospital, Basrah, Iraq; 3 Department of Medicine, University of Basrah, Basrah, Iraq

**Keywords:** retinopathy, diabetes mellitus, camera, Basrah, hyperglycemia

## Abstract

This study aimed to measure the prevalence and risk factors of diabetic retinopathy (DR) among patients with diabetes mellitus aged 20 to 82 years attending the Faiha Diabetes, Endocrine, and Metabolism Center (FDEMC) in Basrah. A cross-sectional study was conducted at FDEMC, including 1542 participants aged 20 to 82 from January 2019 to December 2019. Both eyes were examined for evidence of DR by a mobile nonmydriatic camera, and statistical analysis was performed to measure the prevalence rates (95% CI) for patients with different characteristics. The mean age of participants was 35.9, with 689 males (44.7%; 95% CI: 42.2–47.2%) and 853 females (55.3%; 95% CI: 52.8–57.8%). The prevalence rate of DR was 30.5% (95% CI: 28.1–32.8%), and 11.27% of cases were proliferative retinopathy. DR significantly increased with age (p-value=0.000), it was higher in females (p-value=0.005), and significantly increased with a longer duration of diabetes (p-value<0.001), hyperglycemia (p-value<0.001), hypertension (p-value=0.004), dyslipidemia (p-value<0.001), nephropathy (p-value<0.001) and smoking (p-value<0.001). There was no statistical association between DR and the type of diabetes or obesity. One-third of the participants in this study had DR. Screening and early detection of DR using a simple tool such as a digital camera should be a priority to improve a person’s health status.

## INTRODUCTION

Uncontrolled diabetes mellitus (DM) can lead to a condition called diabetic retinopathy (DR), which affects the blood vessels in the retina of the eye. DR is a major clinical representation of uncontrolled diabetes mellitus and a leading cause of blindness among people with diabetes. The severity of DR is often influenced by the duration of the disease and the level of glucose control [1,2]. Unfortunately, the number of diabetes cases is projected to increase worldwide from 382 million in 2013 to 592 million by 2035 [3]. DR has been identified as the fifth most frequent cause of avoidable blindness and the fifth most frequent cause of moderate to severe visual impairment between 1990 and 2010 [4].

The relationship between hyperglycemia and microvascular complications is not fully understood [5,6]. However, cellular changes can lead to microvascular damage, increased capillary permeability, vascular occlusion, and weakening of supporting structures [1]. In addition, vascular endothelial growth factor (VEGF), which is the basis for treating disorders that could impair vision, may promote the formation of new blood vessels and contribute to vascular leakage [1].

The Early Treatment Diabetic Retinopathy Study (ETDRS – the modified Airlie House classification) classified diabetic retinal disease into two types: non-proliferative diabetic retinopathy (NPDR) and proliferative diabetic retinopathy (PDR) [6,7].

The following are known risk factors for diabetic retinopathy:

- Duration of diabetes mellitus: The Wisconsin Epidemiologic Study of Diabetic Retinopathy (WESDR) found a direct association between the duration of diabetes and the prevalence of DR, which can reach up to 99% and 60% in type 1 and 2 diabetes, respectively, after 20 years. Proliferative diabetic retinopathy represents 50% of type 1 diabetes cases after 20 years and 25% of type 2 diabetes cases after 25 years [8-15].
- Glycemic control: Intensive and early glycemic control (HbA1c<7%) decreases the risk of development or progression of DR in both type 1 DM [16] and type 2 DM [17].
- Hypertension: Tight control of blood pressure (140/80 mmHg) decreases the risk of progression of DR [17,18] and induces a 34% risk reduction in microvascular changes [19]. For every 10mmHg reduction in systolic blood pressure, there was a 13% reduction in microvascular endpoints[19].
- Diabetic kidney disease: The deterioration or treatment of kidney diseases is associated with the worsening or improvement of DR, respectively [20,21]. Most patients with renal disease, as evidenced by proteinuria and/or elevated serum creatinine, have some degree of retinal changes. On the other hand, 35% of asymptomatic retinopathy patients have diabetic kidney disease[19].
- Dyslipidemia: Elevated serum lipids are strongly correlated with worsening DR [22,23].
- Obesity: Some studies have shown that obesity is associated with DR [24].
- Smoking: Smoking increases blood levels of carbon monoxide, platelet aggregation, and vasoconstriction, all of which can increase the risk of diabetic retinopathy [19,25]. However, there is some controversy about the association between smoking and the progression of DR [26, 27].

Patients with diabetes often experience poor visual acuity due to various retinal signs, which can be visualized and recorded using fundus photography - a baseline tool for diagnosing and monitoring retinal diseases. Recently, the introduction of mobile nonmydriatic fundus cameras has greatly improved the quality of DR screening and follow-up programs. This technology is part of telemedicine, which allows patients with diabetes to have their retinas examined at a location outside of a specialized eye examination unit, such as a diabetes center [28-31]. Fluorescein angiography, optical coherence tomography (OCT), and B-scan ultrasonography are other tools used to diagnose DR [6,31,32].

It is essential to differentiate hypertensive retinopathy and other diseases from DR [19]. Imperative management includes controlling associated risk factors and blood glucose to prevent the onset and progression of DR [1,33,34]. Medical intervention may include the use of fenofibrate [35] and intravitreal anti-vascular endothelial growth factors, such as ranibizumab, which is now widely used to treat macular edema [36,37]. Other management options include laser photocoagulation [6,32,38,39] and surgical treatment, such as pars plana vitrectomy [6,32,40].

This study aimed to assess the prevalence of diabetic retinopathy (DR) and its risk factors among patients with diabetes who were receiving care at the Faiha Diabetes, Endocrine, and Metabolism Center in Basrah, located in Southern Iraq.

## MATERIAL AND METHODS

### Study design and setting

This cross-sectional study assessed DR prevalence and risk factors among diabetic patients attending the Faiha Diabetes, Endocrine, and Metabolism Center (FDEMC) in Basrah. The study was conducted at FDEMC from January 2019 to December 2019. A non-random sample was collected by a simple randomization technique, which consisted of 1542 diabetic patients aged 20 to 80 years. Well-trained medical staff recorded electronic data related to clinical and laboratory tests and demographic measures, followed by a fundoscopic examination of the retina using a nonmydriatic mobile camera. All data and retinal images were uploaded to the FDEMC intranet computers and analyzed by groups of endocrinologists and by a single ophthalmologist, respectively.

### Data collection and examinations

For the study requirements, a structured questionnaire was formulated that comprised the following:

- Patient name and patient FDEMC ID number;
- Demographic information, including:
  - Age in years was stratified into groups: 20 to <30, 30 to <40, 40 to <50, 50 to <60, 60 to <70, and ≥70;
  - Gender.
- Clinical history, examinations, and laboratory assessments, including:
  - Type of diabetes mellitus (type 1 or type 2);
  - Duration of diabetes mellitus, which is the period between the age of diagnosis and the time of examination, was categorized into groups: <10 years, 10-30 years, and >30 years;
  - Obesity, defined as BMI (body mass index) greater than or equal to 30 mg/m2, calculated as weight (kg) divided by square height (m2);
  - Smoking history, classified as nonsmokers and smokers (current or past smokers were included in the smoking group);
  - Blood pressure was measured by an automated office blood pressure (AOBP) machine in a quiet room and a seated position. Patients were considered hypertensive if the reading was greater than or equal to 140/90 mm Hg or if they were already on anti-hypertension therapy; results below that level indicated no hypertension;
  - Diabetes control, defined as HbA1c% equal to or less than 7%, measured by high-performance liquid chromatography (Varianttm Hemoglobin Testing System; Bio-Rad Laboratories Inc., Hercules, CA, USA);
  - Serum lipid level, obtained from a fasting blood sample and tested for lipid profile by Integra laboratory diagnostics. Dyslipidemia was defined as an abnormal lipid profile when total cholesterol and triglyceride (TG) levels were equal to or above 200 mg/dl and 150 mg/dl, respectively, and high-density lipoprotein HDL levels were less than 45 mg/dl;
  - Nephropathy, defined as the presence of microalbumin in the urine within the range of 30-300 mg/g of creatinine. The immunoturbidometric assay method is often used to quantify albumin concentrations in urine.
- Ophthalmological examinations: a detailed anterior segment of both eyes was examined, including visual acuity recording, intraocular pressure measurement, and lens opacity examination. A retinal examination was then performed using a nonmydriatic digital retinal camera (D-EYE Portable Retinal Imaging System, d-eye S.r.L.^®^ Padova PD, Italy), which was easily attached to a smartphone (iPhone 6^®^), creating a handheld direct ophthalmoscope for vision care screening and evaluation with no mydriatic eye drops used. The camera used a magnetic fundus lens attached to an iPhone and utilized a user-friendly smartphone application and the built-in iPhone camera to take fundus photographs for each patient. Multiple images were taken for each eye centered on the fovea (450), and these images were graded into nonproliferative DR and a proliferative DR by an ophthalmologist according to Early Treatment Diabetic Retinopathy Study (ETDRS).

The study excluded individuals under 20 years old, with gestational diabetes mellitus, and ocular media opacity (such as cataracts or corneal opacities) that could interfere with proper fundus examination, urinary tract infections, or abnormal thyroid function.

### Statistical analysis

Statistical Package for Social Sciences (SPSS) version 20 was used to analyze qualitative and quantitative data. Age was treated as a quantitative variable. The prevalence rate of DR was calculated with a 95% confidence interval (CI) and compared across different characteristics. A Chi-square test was performed to compare the variables with and without DR. Multiple regression analysis was also analyzed at 95% CI to evaluate the relationship between risk factors and DR. P-values ≤0.05 were considered statistically significant.

## RESULTS

### Prevalence of DR

A total of 1542 patients with a mean age of 35.9 years (range 20-82 years) participated in this study. Of these, 689 were males (44.7%; 95% CI: 42.2%-47.2%), and 853 were females (55.3%; 95% CI: 52.8%-57.8%). 470 patients had DR, resulting in an overall prevalence rate of 30.5% (95% CI: 28.1% to 32.8%), with 11.27% having proliferative changes.

**Figure 1:**
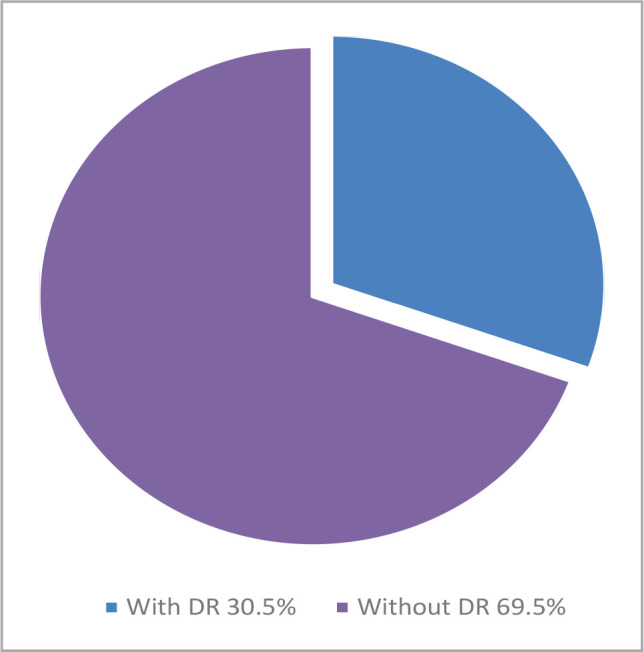
Prevalence of DR in the study population.

Table 1 summarizes the frequency and prevalence of DR with corresponding 95% confidence intervals (CI). Figure 1 shows the prevalence of DR in those patients.

**Table 1:** Frequency and prevalence of DR with corresponding 95% confidence intervals (CIs).

Variable		Frequency		Prevalence		95% Confidence Interval	
						Lower	Upper
**With DR**	Preproliferative	470	417	**30.5%**	88.73%	28.1%	32.8%
	Proliferative		53		**11.27%**		
**Without DR**		1072		69.5%		67.2%	71.9%
**Total**		1542		100.0%		100.0%	100.0%

DR - Diabetic Retinopathy.

Table 2 summarizes the frequency and prevalence of DR among different risk factors with corresponding p-values.

**Table 2:** The frequency and prevalence of DR across different risk factors with corresponding p-values.

Variable				With DR		Without DR		Total		P-value
				Frequency	Percent	Frequency	Percent	Sum	Percent	
**Age (years)**	20–29			15	**1.0%**	92	6.0%	107	7.0%	0.000
	30–39			29	1.9%	144	9.3%	173	11.2%	
	40–49			94	6.1%	305	19.8%	399	25.9%	
	50–59			172	**11.2%**	306	19.8%	478	31.0%	
	60–69			133	8.6%	202	13.1%	335	21.7%	
	≥70			27	1.7%	23	1.5%	50	3.2%	
**Gender**	Male			212	**13.8%**	477	30.9%	689	44.7%	0.043
	Female			258	**16.7%**	595	38.6%	853	55.3%	
**Duration**	<10 years	Pre-PDR	153	162	10.5%	738	47.9%	900	58.4%	0.000
		PDR	9							
	10–29 years	Pre-PDR	237	276	**17.9%**	318	20.6%	594	38.5%	
		PDR	**39**							
	≥30 years	Pre-PDR	27	32	2.1%	16	1.0%	48	3.1%	
		PDR	5							
**Glycemic control**	Uncontrolled	Pre-PDR	365	413	**26.8%**	882	57.2%	1295	84.0%	0.001
		PDR	**48**							
	Controlled	Pre-PDR	52	57	3.7%	190	12.3%	247	16.0%	
		PDR	5							
**DM type**	Type 1	Pre-PDR	28	37	2.4%	151	9.8%	188	12.2%	0.000
		PDR	9							
	Type 2	Pre-PDR	389	433	**8.1%**	921	59.7%	1354	87.8%	
		PDR	44							
**Blood pressure**	Hypertensive	Pre-PDR	279	312	**20.3%**	523	33.9%	835	54.2%	0.000
		PDR	**33**							
	Non-hypertensive	Pre-PDR	138	158	10.2%	549	35.6%	707	45.8%	
		PDR	20							
**Renal disease**	Nephropathy	Pre-PDR	122	151	9.8%	179	11.6%	330	21.4%	0.000
		PDR	**29**							
	Non-nephropathy	Pre-PDR	295	319	**20.7%**	893	57.9%	1212	78.6%	
		PDR	24							
**Lipid profile**	Dyslipidemia	Pre-PDR	280	316	**20.5%**	532	34.5%	848	55.0%	0.000
		PDR	**36**							
	Normal	Pre-PDR	137	154	10.0%	540	35.0%	694	45.0%	
		PDR	17							
**Obesity**	Obese	Pre-PDR	219	246	**16.0%**	551	35.7%	797	51.7%	0.042
		PDR	**27**							
	Non-obese	Pre-PDR	198	224	14.5%	521	33.8%	745	48.3%	
		PDR	26							
**Smoking**	Smoker	Pre-PDR	88	115	7.5%	227	14.7%	342	22.2%	0.019
		PDR	27							
	Non-smoker	Pre-PDR	329	355	**23.0%**	845	54.8%	1200	77.8%	
		PDR	26							
**Total**	**Sum/percent**			**470**	**30.5%**	**1072**	**69.5%**	**1542**	**100%**	

DR - Diabetic Retinopathy; PreDRP - PreProliferative Diabetic Retinopathy; PDR - Proliferative Diabetic Retinopathy.

### Age

Most participants (78.6%) were in the 40-69 age range. There was a higher prevalence of DR in the 50-59 age group (11.2%) and a lower rate in the 20 to 29 age group (1.0%). There was no significant difference in the prevalence of DR between the 30-39 age group (1.9%) and the ≥70 age group (1.7%). The prevalence rate of DR was significantly higher with increasing age (p-value < 0.000).

### Gender

The frequency of females was higher than that of males, 853 (55.3%) and 689 (44.7%), respectively. The prevalence rate of DR was significantly higher in females (16.7%) than in males (13.8%) (p-value < 0.043).

### Diabetes duration

900 patients (58.4%) had diabetes for 10 years or less, 594 patients (38.5%) for 10 to 30 years, and 48 patients (3.1%) for more than 30 years. Despite these findings, the prevalence of DR was higher in patients with a DM duration of 10-30 years (17.9%), of whom PDR was found in 39 patients (p-value <0.000).

### Glycemic control

Blood sugar was uncontrolled in a high percentage of patients (84.0%), among whom DR was found in 26.8%, and some had PDR changes detected in 48 patients (p-value <0.001).

### Type of Diabetes Mellitus

1354 participants (87.8%) had T2DM, and 188 had T1D (12.2%). The prevalence rate of DR was significantly higher in T2DM (28.1%) than in T1D (2.4%), and 44 patients with T2DM had PDR changes (p-value< 0.000).

### Blood pressure

Among the study participants, 835 (54.2%) had hypertension. The prevalence of DR was higher among hypertensive patients (20.3%) than diabetic nonhypertensive patients (10.2%). Furthermore, 33 patients with DR had PDR changes, and this difference was statistically significant (p-value < 0.000).

### Nephropathy

1212 (78.6%) participants had no nephropathy. DR changes were seen more often in patients without nephropathy, while PDR occurred in 29 nephropathic patients (p-value<0.000).

### Dyslipidemia

Lipid tests were abnormal in 848 patients (55.0%). DR occurred in 20.5%, compared to 10.0% of reference ranges, and PDR occurred in 36 patients (p-value< 0.000).

### Obesity

797 (51.7%) had obesity, slightly higher than the number of nonobese patients, 745 (48.3%). The prevalence of DR was 16.0% in obese patients, compared to 14.5% in nonobese patients, with an approximately equal number of patients with PDR changes in both groups, 27 obese patients and 26 nonobese patients (p-value<0.042).

### Smoking

There were 1200 (77.8%) nonsmoker patients and 342 (22.2%) smoker patients, and the prevalence of DR was higher in nonsmoker patients (23.0%) than smoker patients (7.5%), with an approximately equal number of patients with PDR changes in both groups, 27 smoker patients and 26 nonsmoker patients (p-value < 0.019).

### Multivariate analysis

A standard multiple regression was conducted to evaluate the relationship between the identified risk factors and DR, revealing that R2 was 0.610 and the F factor was 239, indicating a significant relationship (p-value=.000). However, gender, obesity, and smoking were not significant predictors of DR (p-value =.412, .367 and .076, respectively).

**Figure 2:**
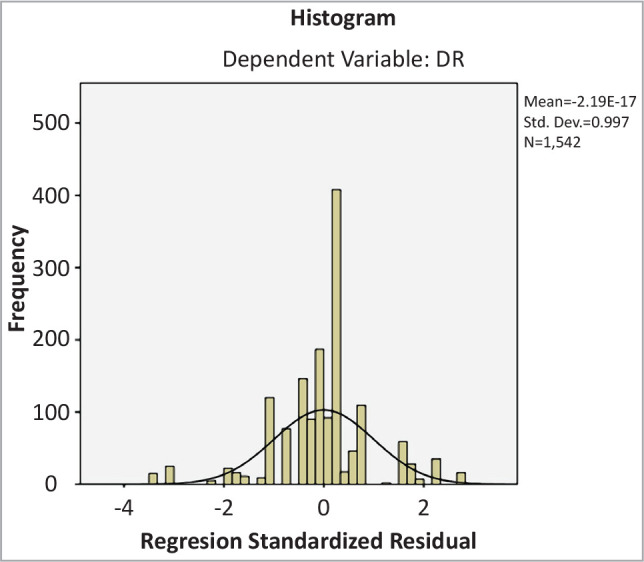
Histogram of DR as the dependent variable.

When analyzing the correlation coefficient, smoking was strongly associated with DR and reduced the association with the type of diabetes (p-value .500), as shown in Table 3 and Figure 2.

**Table 3:** Multiple regression analysis of the risk factors associated with diabetic retinopathy.

Variable	Diabetic Retinopathy (DR)				
	Correlation coefficient				
	Correlations	P-value	B	Std. Error	P-value
**Duration**	-.328	.000	-.259	.026	.000
**Type of diabetes**	-.087	.000	-.028	.042	**.500**
**Age**	-.209	.000	-.453	.021	.000
**Sex**	.006	**.412**	-.095	.034	.005
**Control**	.070	.003	.576	.028	.000
**HT**	.163	.000	.242	.084	.004
**Lipid**	.163	.000	.450	.085	.000
**Renal**	.173	.000	.593	.037	.000
Obesity	.009	**.367**	-.001	.040	**.974**
**Smoking**	.036	**.076**	.246	.040	.000
**R2**	.610				
**F factor**	239.669; P-value .000				

## DISCUSSION

The increasing incidence of diabetes worldwide is a significant global healthcare concern, with an estimated 415 million people (age 20-79 years) currently living with diabetes, of whom approximately 50% remain undiagnosed. Diabetic retinopathy is a common complication of untreated diabetes that can progress to visual impairment and blindness [41]. Generally, the probability of blindness in a person with diabetes is 25 times greater than that in the general population; hence early detection of DR is the first step in preventing vision loss. The American Academy of Ophthalmology and the American Diabetes Association suggest that annual ophthalmic examinations should start from the day of a diabetes diagnosis. However, in the past, the lack of accessible and efficient tools to detect early retinal changes led to delayed DR diagnosis and increased healthcare burden [42].

This study detected DR in 30.5% (95% CI: 28.1% to 32.8%) of patients aged 20 years and above attending FDEMC in Basrah (southern Iraq). Of these patients, 11.27% had proliferative changes. The prevalence rate of DR in other governorates of Iraq was consistent with our study. In two studies conducted in Baghdad, the prevalence rates of DR were 30.2% [43] and 33.1% [44]. One study aimed to assess the prevalence rate and risk factors of visual acuity, retinopathy, cataracts, and glaucoma among a large sample size of diabetic patients aged 20-65 years, which differs from the objective of our study. In the other study, the sample size was small, and they assessed the importance of insulin therapy with the other risk factors. A study conducted in Mosul on proliferative and nonproliferative DR of T1D and T1DM had a small sample size and did not include the risk factors mentioned in our study. However, the prevalence rate was 32.35%[45]. Other studies in the region around Iraq reported variable rates of DR, including Jordan (34.1%)[46], Turkey (42.8%)[47], Saudi Arabia (36.4%)[48], Iran (41.9%)[49], Oman (42.4%)[21], United Arab Emirates (19%)[20], Qatar (23.5%)[50], Egypt (20.5%)[51] and Lebanon (35%)[52].

The global prevalence rates of DR and proliferative DR among patients with diabetes are 35.4% and 7.5%, respectively [4], confirmed in a pooled meta-analysis of 35 studies from 1980-2008 and consistent with our current study.

Internationally, the prevalence rate of DR was studied in different countries, including European countries, with diverse rates ranging from 4% in Finland to 52% in the UK, with an average of nearly 40%[53]. Variations due to race- and ethnicity-related differences in the prevalence of DR have been considered an important public health issue. Similar variations have also been reported in the USA, ranging from 3.1% to 48% among ethnic groups [53]. Other countries reported DR prevalence rates as follows: the Russian Federation (34.2%)[53], China (27.9%)[54], Korea (15.8%)[55], Singapore (28.2%)[56], and Australia (35.5%)[53].

In our study, there was a relationship between DR and age group, with higher prevalence occurring in the 50-59 age group (11.2%). These findings are consistent with previous studies [20, 44-46, 48, 51, 57-60].

The association of sex with the development of DR is controversial. Various studies have reported that men are at higher risk than women [20, 58, 61], whereas other studies found no gender differences [46, 48, 55, 59, 62] or a higher risk for women[45, 51]. In our study, women had a greater risk of DR development than men (16.7% and 13.8%, respectively).

The duration of diabetes in our study was strongly associated with DR, especially at 10-29 years (17.9%), and DR in patients with a duration greater than 30 years was less common (2.1%), which was due to a lower number of participating patients in those groups (3.1%). These results were consistent with other studies [20, 21, 43-46, 48, 51, 52, 54, 55, 57, 58, 61,63,64]. However, in other studies, the opposite was identified [59].

In most previous studies, hyperglycemia (measured by HbA1c) was considered a significant risk factor for the development of DR [43-46,48, 50, 54, 55, 61, 64], which is consistent with the results of our study. However, other studies did not report these findings [51, 59, 63].

Type 1 diabetes usually has a higher risk of retinopathy due to the longer duration of the disease [20, 51, 64]. Nevertheless, we did not see these results in our study, where T2DM was more commonly associated with DR than T1D in univariate analysis, possibly due to the high participation rate of patients 20 years old and above. Other studies have reported equal rates of DR in both types of diabetes [45], which was shown by multivariate analysis.

Hypertensive patients with diabetes were considered a distinctive risk factor for DR in our study, and those results were consistent with other studies [20, 24, 43-45, 48, 51, 52, 54, 55, 57, 61,63,64].

Nephropathy was not a risk factor for DR in our study in univariate analysis. However, there was a significant relationship between nephropathy and DR in the multivariate analysis, and these results were consistent with other studies [20, 48, 52, 54, 63].

There was a strong association between dyslipidemia in diabetic patients and the development of DR in our study, which was proven in several studies [22-24, 44, 54, 57, 59, 63]. These results were not consistent with other studies [65].

Our study demonstrated that obesity was not a risk factor for DR, consistent with other studies [55, 57] but not reported in others [24, 44, 59].

The relationship between smoking and DR development is controversial, as the relationship in our study was negative, which is consistent with other findings [26,27,46,57]. However, other studies have reported the opposite. When analyzed by multiple regression tests, smoking had a strong association with DR, in line with other studies [19, 25, 43].

The risk factors under study play a role in the development of DR apart from obesity and type of diabetes. At first, smoking had a weak association with DR, but regression analysis [26] removed the factors that caused the relationship to be underestimated. It is evident from Table 3 that these risk factors explain 61% of the development of DR, leaving approximately 40% affected by other factors not included in our study, and hyperglycemia is part of the disease process.

To date, no published Basrah hospital-based studies have addressed retinopathy in diabetic patients despite its negative effect on the quality of life. The retinopathy screening program has not been fully established in our region. For this reason, the use of nonmydriatic digital cameras, as in other countries [29, 30, 66-70], may reduce the time and effort in the early detection of retinopathy. The easy-to-use nonmydriatic digital camera allows physicians and ophthalmologists to screen for retinopathy efficiently and intervene at an early stage, potentially preventing progression to more advanced disease, which is what we tried to prove in this study.

This study holds significant importance as it utilized a large sample size to determine the prevalence of diabetic retinopathy (DR) while prospectively evaluating diabetic patients. This approach enables early referral to a retinal disease specialist, highlighting the importance of timely detection and management of DR. Additionally, this study addressed a group of risk factors associated with DR using modern screening methods. Furthermore, all data for this study have been uploaded via an internet network with easy access by endocrinology specialists for review and analysis. In addition, we assessed retinal changes using a nonmydriatic digital camera, which provided us with 450 fundus images that were ready to be analyzed by an ophthalmologist.

There are several limitations to this study that must be considered. First, the study was conducted in a single center, and as a result, the findings cannot be generalized to the broader population. Additionally, the retinal images were interpreted by a single ophthalmologist, which increases the risk of bias. Second, this is a cross-sectional study in which DR prevalence data and risk factor information were derived concurrently rather than a case-control or cohort study. Therefore, the results must be interpreted with caution. Smoking behavior was derived from questionnaires rather than objective measurements; thus, more than three-quarters of diabetic patients in our study were nonsmokers, which affects the estimation of the prevalence rate of DR and the causal association. Finally, the use of a digital camera for retinal changes improves sensitivity and specificity compared to standard fundoscopy [69], but it has a limited ability to detect peripheral lesions.

## CONCLUSION

DR was observed in one-third of patients, with one-tenth of those cases being proliferative. Our findings indicate that increasing age, female gender, longer diabetes duration, hyperglycemia, hypertension, dyslipidemia, nephropathy, and smoking had a direct correlation with DR. On the other hand, other risk factors, such as type of diabetes and obesity, had no proven effect. The use of nonmydriatic digital cameras has demonstrated the potential to overcome obstacles in the early diagnosis of DR. Nevertheless, future studies involving larger sample sizes, including the Basrah population, are needed to confirm these findings.
